# Supplementary figures and images for: Adaptation of the periplasm to maintain spatial constraints essential for cell envelope processes and cell viability
Source: eLife. 2022 Jan 27;11:e73516. doi: 10.7554/eLife.73516 (PMC8824477; doi:10.7554/eLife.73516)

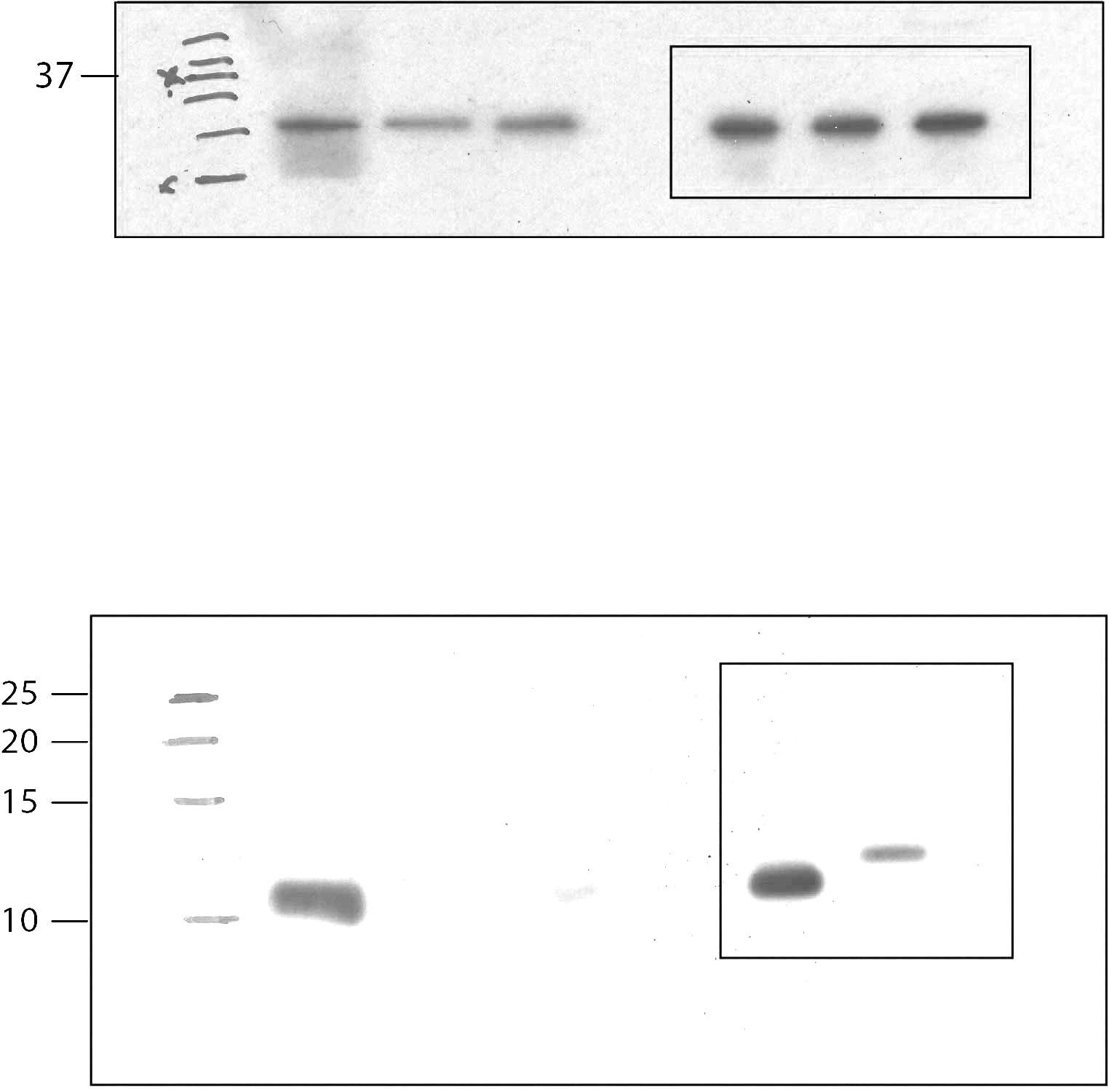

Supplement: Source data 1. [file elife-73516-supp7.zip › Source_data/Figure_1B-Source_data.jpg]

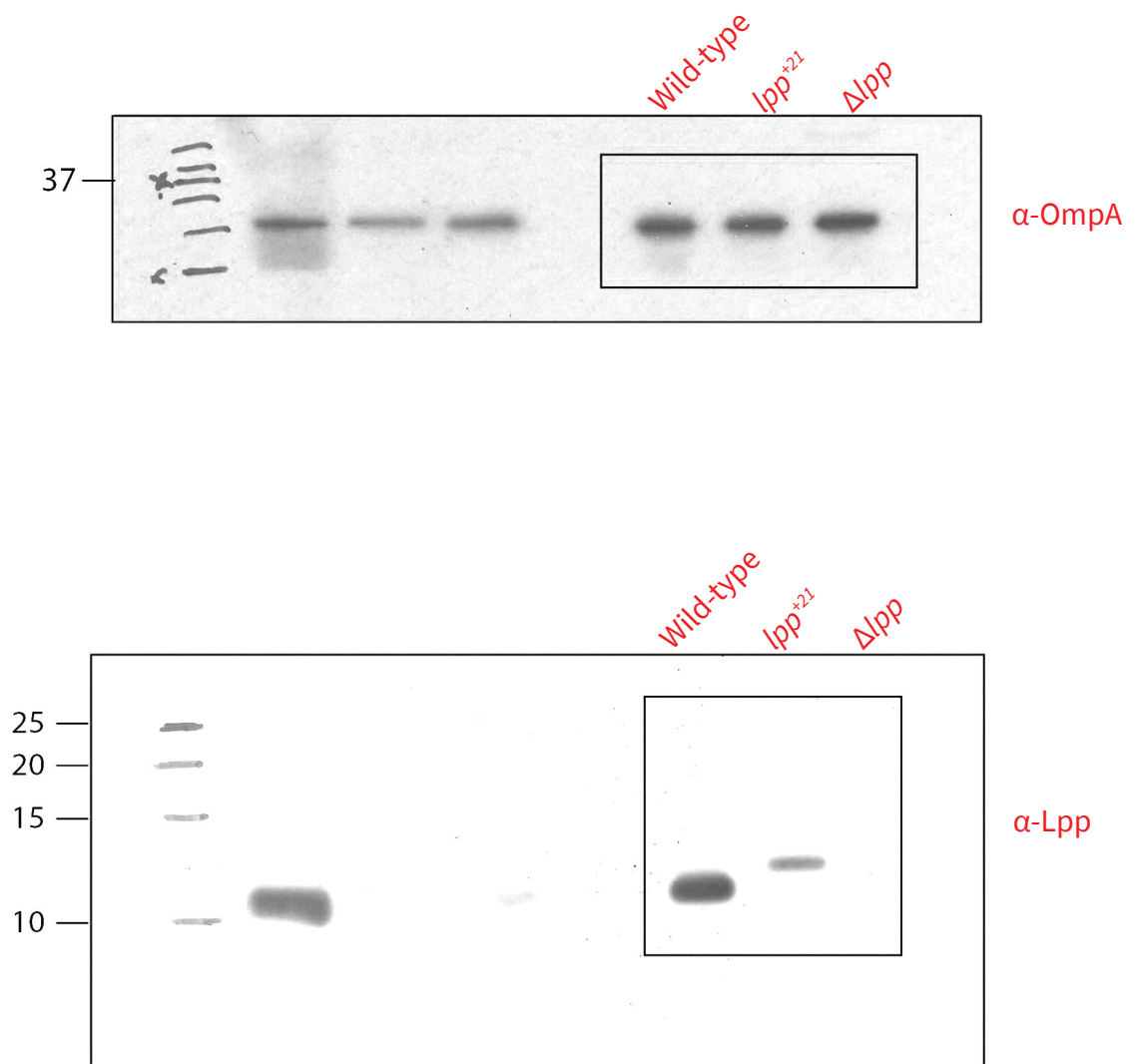

Figure 1B Source Data

Supplement: Source data 1. [file elife-73516-supp7.zip › Source_data/Figure_1B-Source_data.pdf]

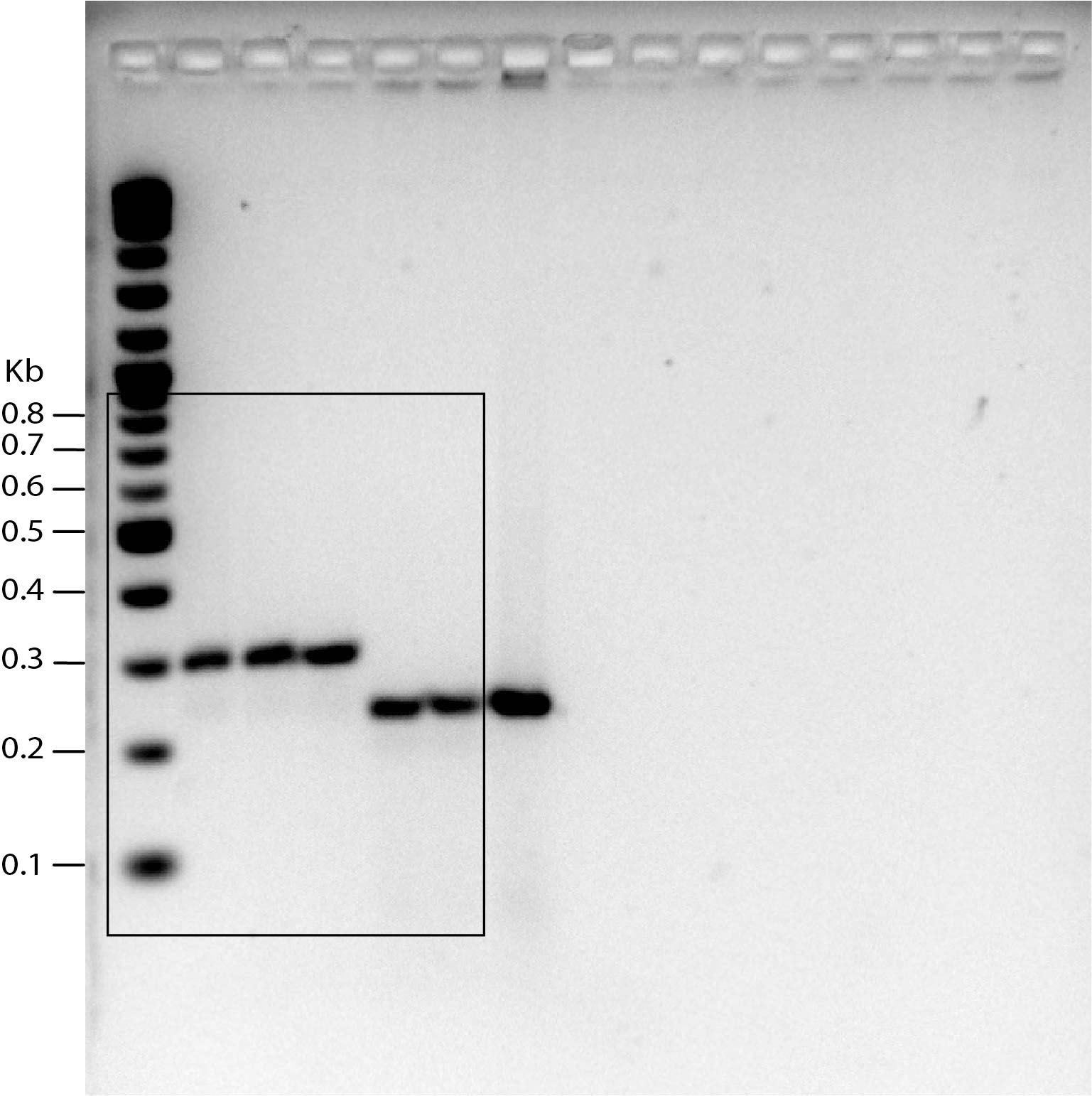

Supplement: Source data 1. [file elife-73516-supp7.zip › Source_data/Figure_S1C-Source_data.jpg]

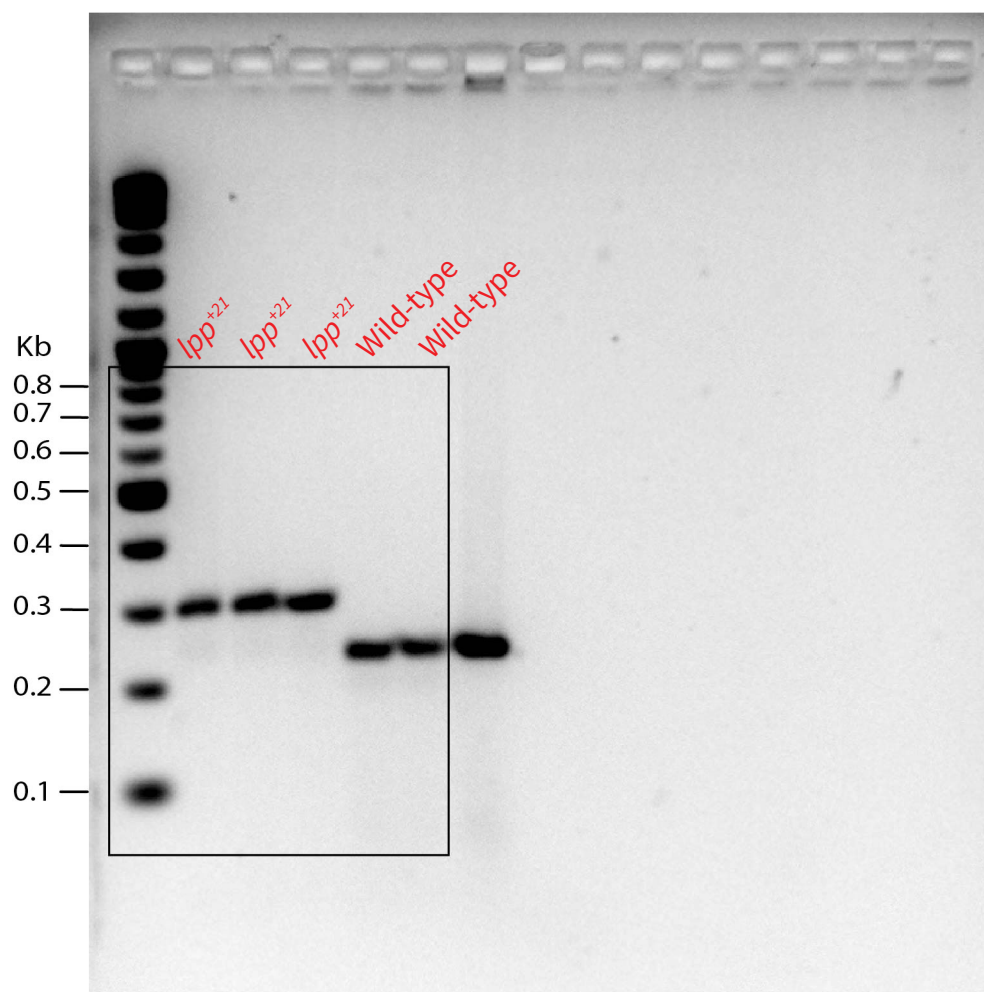

Figure S1C Source Data

Supplement: Source data 1. [file elife-73516-supp7.zip › Source_data/Figure_S1C-Source_data.pdf]

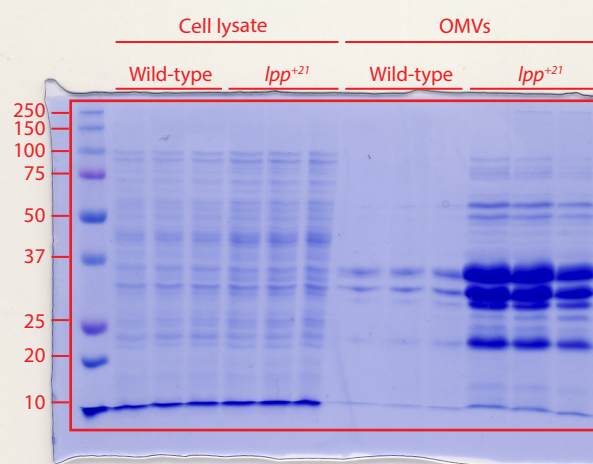

Figure S3B Source data

Supplement: Source data 1. [file elife-73516-supp7.zip › Source_data/Figure_S3B-Source_data.pdf]

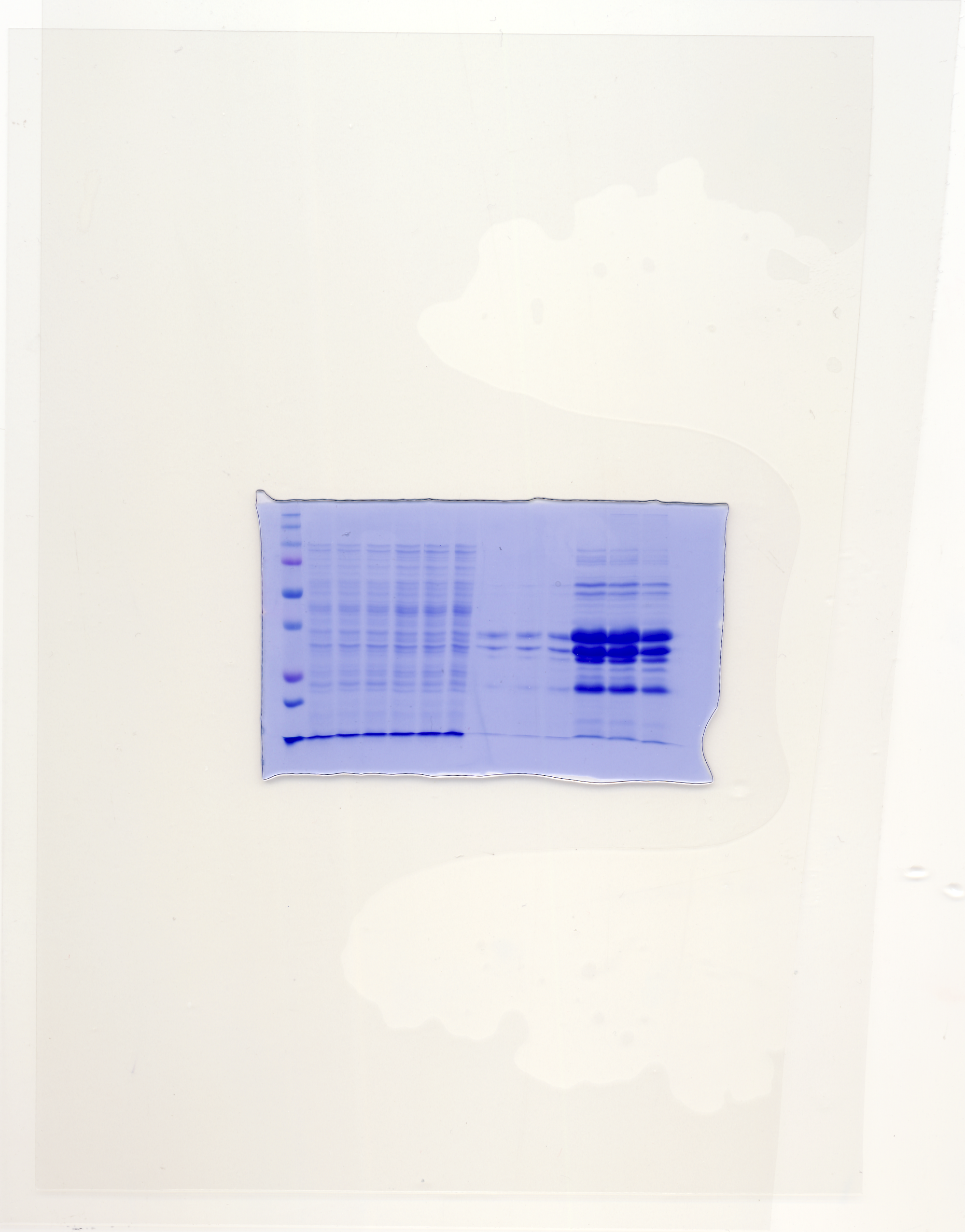

Supplement: Source data 1. [file elife-73516-supp7.zip › Source_data/Figure_S3B-source_data.TIF]

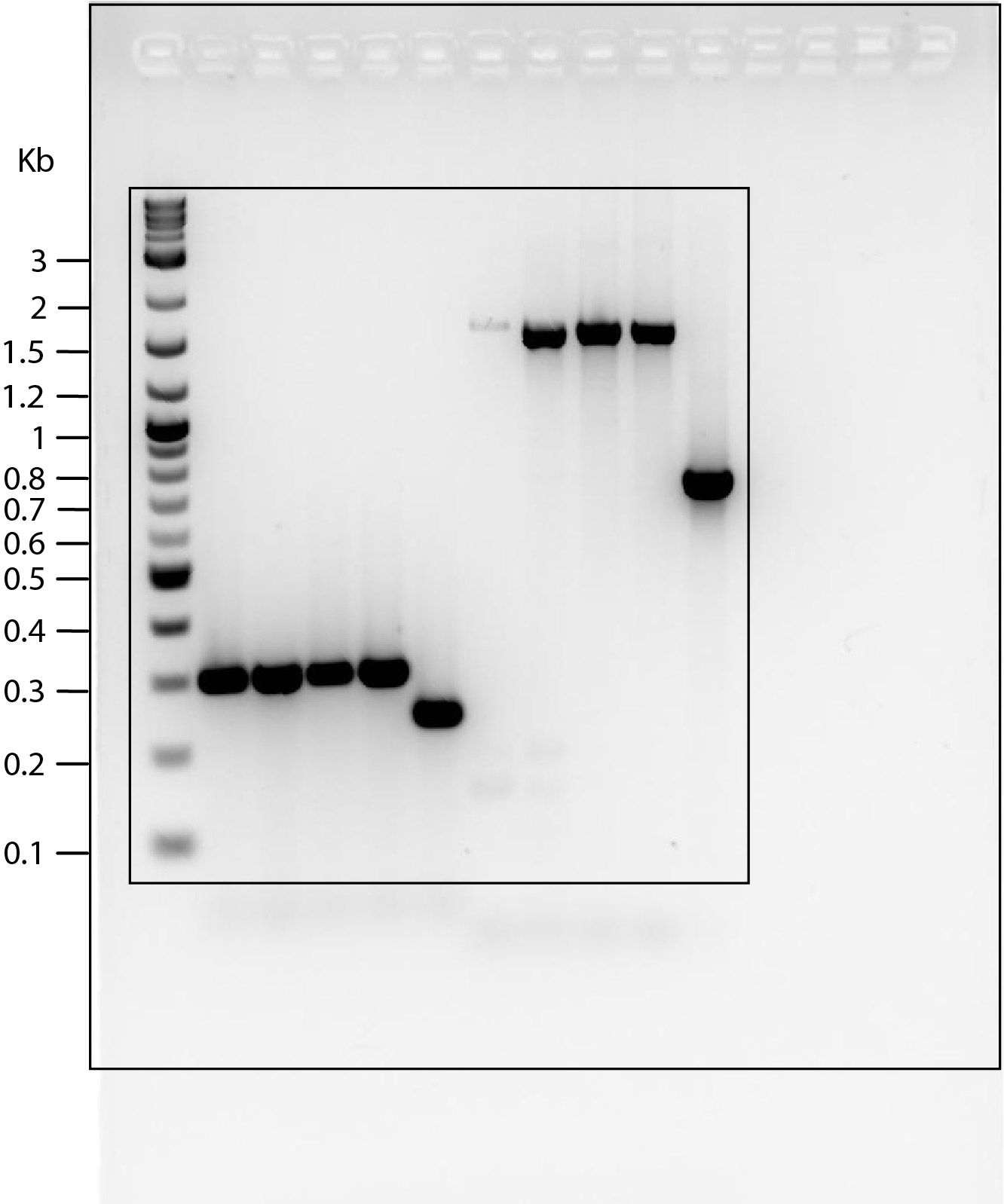

Supplement: Source data 1. [file elife-73516-supp7.zip › Source_data/Figure_S5-Source_data.jpg]

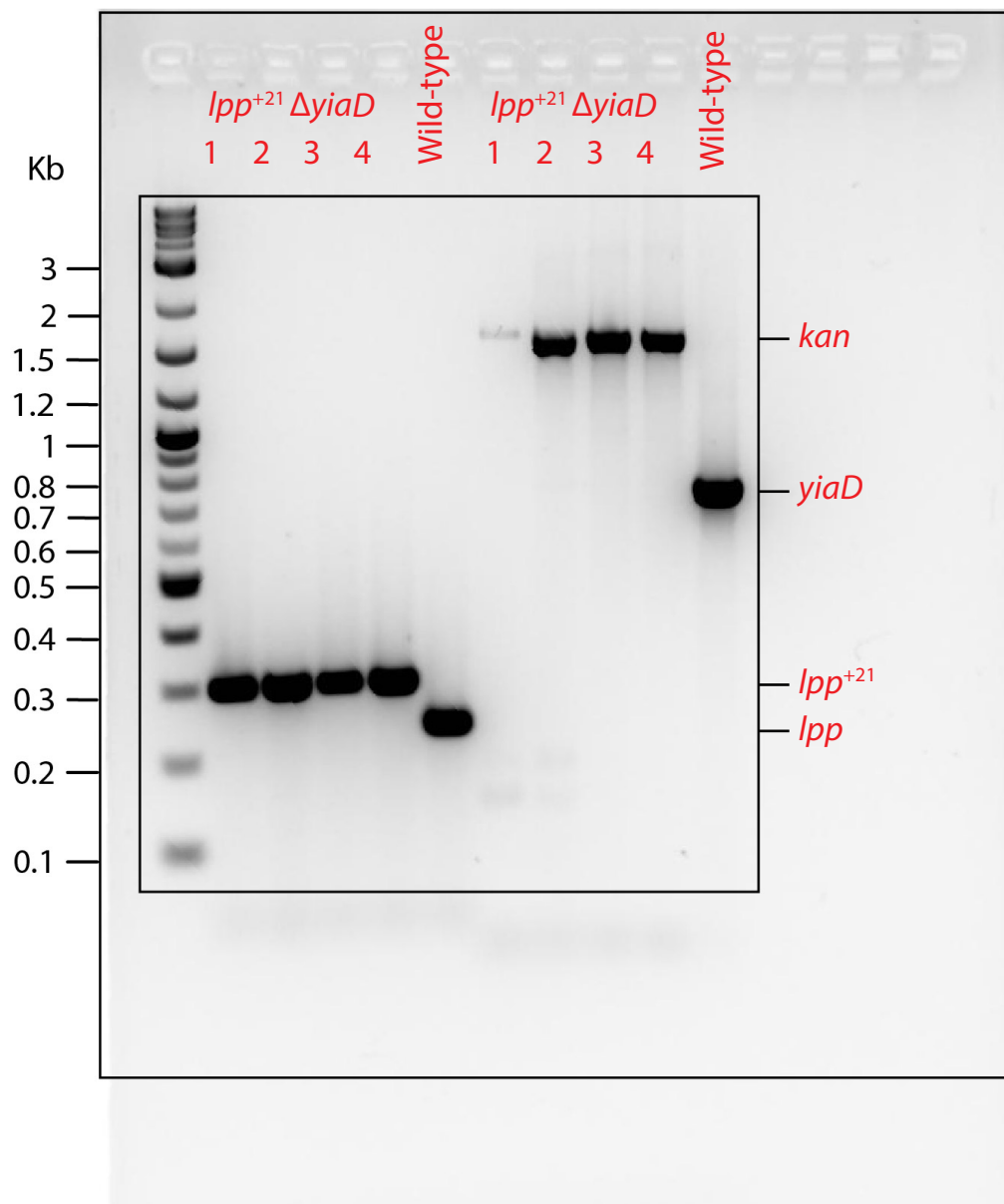

Figure S5 Source Data

Supplement: Source data 1. [file elife-73516-supp7.zip › Source_data/Figure_S5-Source_data.pdf]
